# Association of Remnant Cholesterol With the Retinal Capillary Plexus

**DOI:** 10.1167/tvst.15.4.18

**Published:** 2026-04-16

**Authors:** Shasha Ding, Xiaoxuan Zhu, Kai Yang, Yudie Wang, Zhenqing Wang, Ziyu Li, Wei Lian, Jinxia Yan, Wenya Chen, Mingye Yang, Renjie Zhang, Lulin Lou, Yingjin Li, Wanru Zhao, Yunguang Zhang, Jia Qu, Fan Lu, Lele Cui, Ming Li

**Affiliations:** 1 Eye Hospital, Wenzhou Medical University, National Clinical Research Center for Ocular Diseases, Wenzhou, Zhejiang, China

**Keywords:** remnant cholesterol, dyslipidemia, optical coherence tomography angiography, retinal capillary plexus, atherosclerotic cardiovascular disease

## Abstract

**Purpose:**

Remnant cholesterol (RC) is a well-recognized risk factor for cardiovascular diseases (CVDs) and an important biomarker of cardiovascular risk. However, studies on the effects of RC on the early retinal microvasculature are still scarce. Therefore, this study aimed to investigate the association between RC and the retinal capillary plexus (RCP) using optical coherence tomography angiography (OCTA).

**Methods:**

This cross-sectional study evaluated data from participants of the Jidong Eye Cohort Study. All participants underwent comprehensive ophthalmic examinations and laboratory tests. The RC values were calculated using a recognized formula. OCTA was used to measure vessel density in the RCP.

**Results:**

This study included a total of 3903 participants, with a mean age of 43.42 ± 9.88 years; 1946 were female (49.86%). After adjusting for confounding factors, higher RC levels were significantly associated with lower deep RCP vessel density in the parafovea (β = −0.578 per 1-mmol/L increase; 95% confidence interval, −0.875 to −0.282; *P* < 0.001). Moreover, a significant interaction was observed between sex and RC for deep RCP vessel density in the parafovea (*P* for interaction = 0.029), with a stronger negative association in male participants.

**Conclusions:**

Higher RC levels were significantly associated with lower deep RCP vessel density. Our findings provide evidence supporting the adverse effects of high RC levels on the retinal microvasculature.

**Translational Relevance:**

These findings highlight that RC regulation may help prevent microvascular complications, with implications for clinical strategies targeting microvascular health.

## Introduction

Dyslipidemia refers to unhealthy changes in the plasma lipid profile, often characterized by elevated total cholesterol (TC), low-density lipoprotein cholesterol (LDL-C), or triglycerides (TGs) or reduced high-density lipoprotein cholesterol (HDL-C). Among these, LDL-C is a major risk factor for atherosclerotic cardiovascular disease (ASCVD) and is regarded as the primary target for lipid-lowering therapy.^1^ However, recent studies have shown that significant residual cardiovascular risk persists even when LDL-C levels are controlled within the normal range.^2^^,^^3^ Mounting evidence indicates that remnant cholesterol (RC) may be a critical factor contributing to this residual risk, as triglycerides can be readily metabolized by most cells in the body, whereas their cholesterol components play a critical role in the process of atherosclerosis.^4^ RC is a nontraditional lipid parameter and refers to the cholesterol carried in the remnant particles produced after the lipolytic metabolism of triglyceride-rich lipoproteins (TRLs), which is the cholesterol in hydrolyzed very-low-density lipoproteins and intermediate-density lipoproteins in the fasting state and the cholesterol in the chylomicron remnants in the postprandial state.^5^^,^^6^ Extensive previous studies have demonstrated that RC is significantly associated with an increased residual risk of macrovascular diseases such as ASCVD, and this association is independent of LDL-C.^7^^,^^8^ Additionally, RC is closely linked to the onset and progression of microvascular diseases, including diabetic retinopathy (DR),^9^^,^^10^ with retinal microvascular injury potentially being the earliest detectable change during disease development.^11^^,^^12^ Therefore, it is essential to explore the effects of RC on the retinal microvasculature.

The retina is the only terminal branch of the cardiovascular system that can be directly visualized in the human body, providing a unique window for assessing microvascular health.^13^^–^^15^ Previous studies have reported a strong association between elevated atherogenic lipid levels and retinal arteriolar narrowing in adolescents.^16^ Furthermore, a cross-sectional study of individuals with type 2 diabetes demonstrated that higher remnant cholesterol levels were associated with wider retinal arterioles and venules.^17^ However, conventional fundus photography has significant limitations; restricted by image magnification and resolution, it cannot accurately quantify subtle microvascular changes or detect early microcirculatory abnormalities at the capillary level, potentially leading to the underdiagnosis of subclinical microvascular damage. Optical coherence tomography angiography (OCTA) is a noninvasive imaging technique that enables the quantitative visualization and dynamic monitoring of retinal vascular networks.^18^ This technology not only is widely used in the clinical diagnosis of retinal diseases but also provides critical evidence for early screening of systemic disorders, including hypertension,^19^ diabetes,^20^ neurodegenerative diseases,^21^^,^^22^ and cardiovascular events.^23^^,^^24^ Previous OCTA studies have demonstrated that both hypertensive and diabetic patients exhibit varying degrees of retinal microvascular impairment, including reduced vessel density and an enlarged foveal avascular zone (FAZ) area.^25^^–^^27^ However, the effect of RC on subtle changes in vessel density within the retinal capillary plexus (RCP) remains unclear. Therefore, this study aimed to investigate the association between RC and the RCP using OCTA in a large community-based population.

## Materials and Methods

### Study Design and Participants

**Figure 1.**
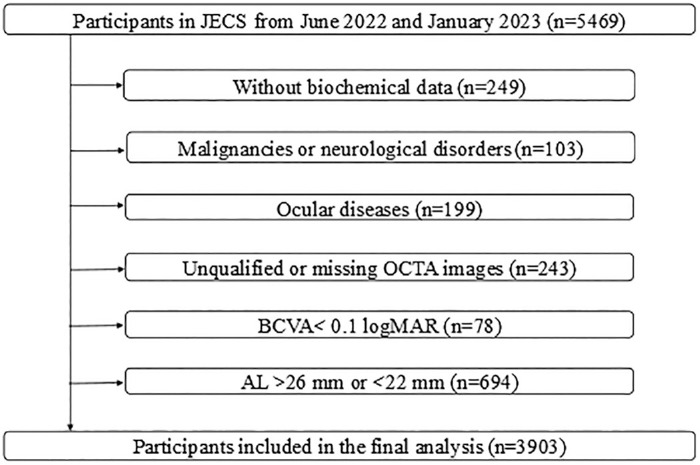
Flow chart of the study participants.

The study participants were all recruited from the Jidong Eye Cohort Study (JECS), a prospective cohort study among the community population in Jidong communities (Tangshan, Hebei, China). The research design has been previously described in detail.^28^ A total of 5469 participants who completed comprehensive examinations were recruited between June 2022 and January 2023. After the exclusion of 249 participants without biochemical data, 103 with malignancies or neurological disorders, 199 with ocular diseases, 243 with unqualified OCTA images, 78 with best-corrected visual acuity (BCVA) worse than 0.1 logMAR (Snellen visual acuity <20/25), and 694 persons with an axial length (AL) > 26 mm or < 22 mm, the final analysis included 3903 eligible participants (Fig. 1).

This study followed the tenets of the Declaration of Helsinki and was approved by the Ethics Committee of Jidong Oilfield Hospital of PetroChina (No. 2018 YILUNZI1) and the Ethics Committee of Wenzhou Medical University Affiliated Ophthalmology Hospital (2021-074-K-63-01). All participants voluntarily provided written informed consent.

### Assessment of General Variables

In this study, participants’ baseline characteristics were collected through laboratory tests, clinical examinations, and demographic/lifestyle questionnaires.^29^ Body mass index (BMI) was calculated as weight (kg) divided by height squared (m^2^). Education level was categorized into “illiteracy, elementary school, junior high school, or high school” or “college or above.” The average monthly income was classified as ≤¥3,000 or >¥3,000. Hypertension was defined as a systolic blood pressure (SBP) ≥ 140 mm Hg, a diastolic blood pressure (DBP) ≥ 90 mm Hg, a self-reported history of hypertension, or the current use of antihypertensive medications. Diabetes mellitus was defined as a fasting blood glucose (FBG) concentration ≥ 7.0 mmol/L, a self-reported history of diabetes, or the current use of glucose-lowering medications. Cardiovascular disease (CVD) was defined as a self-reported history of coronary heart disease, myocardial infarction, or stroke.

### Assessment of Remnant Cholesterol

Venous blood samples were collected from the antecubital vein after an overnight fast (≥8 hours of fasting and no water intake) and preserved in ethylenediaminetetraacetic acid (EDTA)-anticoagulated vacuum tubes. The samples were immediately transported to the central laboratory of Jidong Oilfield Hospital for analysis. Biochemical indicators, including TC, TGs, HDL-C, and LDL-C, were measured using an autoanalyzer (Hitachi, Tokyo, Japan). RC levels were calculated using the following formula: RC = TC − HDL-C − LDL-C. Participants were categorized into quartiles based on RC levels: Q1, RC < 0.68 mmol/L; Q2, 0.68 mmol/L ≤ RC < 0.88 mmol/L; Q3, 0.88 mmol/L ≤ RC < 1.10 mmol/L; and Q4, RC ≥ 1.10 mmol/L.

### Ophthalmic Examination

All participants underwent standardized ophthalm-ic examinations. BCVA was measured at a 5-meter distance using a standard logarithmic visual acuity chart. Refractive status was assessed using an autorefractor (KR-800; Topcon, Tokyo, Japan), with the results expressed as spherical equivalents (SEs; spherical plus half of the cylinder). AL was assessed using an optical biometer (Lenstar LS 900, Haag-Streit, Koeniz, Switzerland). Intraocular pressure (IOP) was determined using a noncontact tonometer (TX-20; Canon, Tokyo, Japan). Anterior segment evaluation was performed using slit-lamp biomicroscopy, and posterior segment assessment was performed using a Canon digital fundus camera. All of the examination results were independently reviewed by two ophthalmologists, and any diagnostic discrepancies were resolved by a third senior ophthalmologist. Participants with ocular conditions such as diabetic retinopathy, glaucoma, traumatic ocular injury, keratoconus, or retinal breaks were excluded from the analysis.

### OCTA Examination

The retinal microvasculature was imaged using spectral-domain OCTA (RTVue XR Avanti with AngioVue; Optovue, Fremont, CA). OCTA images were obtained with a 3 × 3-mm^2^ scan pattern, with a scan density of 304 × 304 A-scans. The RCP was automatically segmented into the superficial RCP and deep RCP using built-in software (AngioAnalytics 2017.1.0.15). The superficial RCP extends from the internal limiting membrane to the inner plexiform layer (IPL), and the deep RCP spans from the IPL to the outer plexiform layer. The RCP vessel density was defined as the percentage of the area occupied by flow signals detected by OCTA in each specific retinal layer. In accordance with the Early Treatment Diabetic Retinopathy Study (ETDRS) grid, the measurement area was divided into the foveal zone (1-mm diameter circle) and the parafoveal zone (1- to 3-mm annular ring partitioned into temporal, superior, nasal, and inferior quadrants). Additionally, the FAZ area was extracted using built-in software. Given the FAZ and specific distribution of retinal ganglion cells, the parafoveal region was selected as the representative area in this study, although the results from other regions are also presented.

The OCTA device incorporated a built-in, three-dimensional projection artifact removal algorithm (Optovue) to automatically eliminate motion artifacts in the deep RCP, thus improving image quality. Scans with a signal quality index < 7/10, inaccurate centration, segmentation errors, or significant artifacts were excluded from this study.^30^ The right eye was primarily analyzed; data for the left eye were substituted when the quality criteria for the right-eye images were not met.

### Statistical Analysis

Continuous variables are presented as mean ± SD, and categorical variables are expressed as frequencies and percentages. Mode imputation was applied to categorical variables (current smoking status, current drinking status, education level, and income), and mean imputation was applied to continuous variables (SBP and DBP). One-way analysis of variance (ANOVA) was used to analyze between-group differences for normally distributed continuous variables, and χ^2^ tests or Fisher's exact tests were employed for categorical variables. Multivariable generalized linear models were applied to estimate the association of a 1-mmol/L increase in RC with RCP vessel density. Additionally, the association between RC quartiles and RCP vessel density was evaluated. RC quartiles were treated as a continuous ordinal variable for trend tests. Two models were constructed for each outcome: Model 1 was adjusted for age, sex, current smoking status, current drinking status, hypertension status, diabetes status, BMI, AL, and CVD, and Model 2 was further adjusted for blood lipid profiles (TG, HDL-C, and LDL-C levels). The adjusted models incorporated interaction terms to evaluate whether sex moderates the association between RC and the RCP. The potential modifying effects of hypertension and diabetes on the relationship between RC and the RCP were also evaluated.

The strengths of the associations are presented as β values with 95% confidence intervals (CIs). All of the statistical analyses were two sided, and *P* < 0.05 was considered to indicate statistical significance. All of the statistical analyses were performed using R 4.5.0 (R Foundation for Statistical Computing, Vienna, Austria) and SAS 9.4 (SAS Institute, Cary, NC).

## Results

### Baseline Characteristics

A total of 3903 participants were ultimately included in the analyses. The mean age of the included participants was 43.42 ± 9.88 years, and 1946 were female (49.86%). The baseline characteristics of the different quartiles of the RC level are displayed in Table 1. Individuals with higher RC levels were more likely to be male, were more likely to be smokers and alcohol consumers, had a higher prevalence of hypertension and diabetes, and had worse metabolic profiles characterized by lower HDL-C levels and higher BMI, FBG, TC, and TG levels (all *P* < 0.05). Additionally, individuals with higher RC levels generally had lower levels of education.

**Table 1.** Baseline Characteristics of Participants by RC Level Quartiles

		RC				
Characteristic	Total	Q1	Q2	Q3	Q4	*P*
*N*	3903	976	966	978	983	—
Age (y), mean ± SD	43.42 ± 9.88	43.24 ± 9.00	43.03 ± 9.98	43.52 ± 10.56	43.86 ± 9.91	0.274
Sex, n (%)						<0.001
Female	1946 (49.86)	511 (52.36)	532 (55.07)	515 (52.66)	388 (39.47)	
Male	1957 (50.14)	465 (47.64)	434 (44.93)	463 (47.34)	595 (60.53)	
Educational level, *n* (%)						<0.001
Illiteracy/primary/middle school	846 (21.68)	152 (15.57)	176 (18.22)	253 (25.87)	265 (26.96)	
College/university	3057 (78.32)	824 (84.43)	790 (81.78)	725 (74.13)	718 (73.04)	
Income, *n* (%)						0.139
≤¥3000	243 (6.23)	72 (7.38)	65 (6.73)	57 (5.83)	49 (4.98)	
>¥3000	3660 (93.77)	904 (92.62)	901 (93.27)	921 (94.17)	934 (95.02)	
Hypertension, *n* (%)	925 (23.70)	235 (24.08)	202 (20.91)	226 (23.11)	262 (26.65)	0.027
Diabetes, *n* (%)	360 (9.22)	91 (9.32)	71 (7.35)	70 (7.16)	128 (13.02)	<0.001
Current smoking, *n* (%)	859 (22.01)	187 (19.16)	185 (19.15)	204 (20.86)	283 (28.79)	<0.001
Current drinking, *n* (%)	1189 (30.46)	255 (26.13)	284 (29.40)	287 (29.35)	363 (36.93)	<0.001
BMI (kg/m^2^), mean ± SD	24.58 ± 3.65	24.35 ± 3.73	24.19 ± 3.60	24.57 ± 3.63	25.21 ± 3.54	<0.001
FBG (mmol/L), mean ± SD	5.78 ± 1.31	5.74 ± 1.27	5.67 ± 1.10	5.71 ± 1.18	5.99 ± 1.62	<0.001
DBP (mm Hg), mean ± SD	75.30 ± 11.91	76.22 ± 11.43	74.55 ± 11.96	74.20 ± 12.12	76.21 ± 11.98	<0.001
SBP (mm Hg), mean ± SD	124.42 ± 17.77	125.94 ± 17.48	123.76 ± 17.85	122.58 ± 18.08	125.40 ± 17.48	<0.001
TC (mmol/L), mean ± SD	4.98 ± 0.94	4.70 ± 0.85	4.87 ± 0.88	4.94 ± 0.88	5.42 ± 0.99	<0.001
TG (mmol/L), mean ± SD	1.71 ± 1.57	1.20 ± 0.59	1.37 ± 0.78	1.58 ± 1.06	2.67 ± 2.54	<0.001
LDL (mmol/L), mean ± SD	2.76 ± 0.80	2.84 ± 0.80	2.76 ± 0.82	2.67 ± 0.81	2.75 ± 0.76	<0.001
HDL (mmol/L), mean ± SD	1.29 ± 0.30	1.30 ± 0.29	1.34 ± 0.32	1.29 ± 0.29	1.25 ± 0.29	<0.001
SE (D), mean ± SD	−1.85 ± 2.15	−2.05 ± 2.14	−1.96 ± 2.23	−1.68 ± 2.22	−1.70 ± 2.00	<0.001
AL (mm), mean ± SD	24.13 ± 0.98	24.20 ± 0.97	24.13 ± 1.00	24.06 ± 0.99	24.13 ± 0.96	0.018
IOP (mm Hg), mean ± SD	15.89 ± 2.39	15.92 ± 2.41	15.86 ± 2.32	15.80 ± 2.42	15.97 ± 2.40	0.465
RC (mmol/L), mean ± SD	0.93 ± 0.40	0.56 ± 0.08	0.77 ± 0.06	0.98 ± 0.06	1.42 ± 0.45	<0.001
CVD, n (%)	64 (1.64)	23 (2.36)	14 (1.45)	15 (1.53)	12 (1.22)	0.215

*P* values were calculated by ANOVA for continuous variables and the χ^2^ test for categorical variables. Participants were categorized into quartiles based on RC levels: Q1, RC < 0.68 mmol/L; Q2, 0.68 mmol/L ≤ RC < 0.88 mmol/L; Q3, 0.88 mmol/L ≤ RC < 1.10 mmol/L; Q4, RC ≥ 1.10 mmol/L.

### RCP Vessel Density in RC Quartile Groups

**Figure 2.**
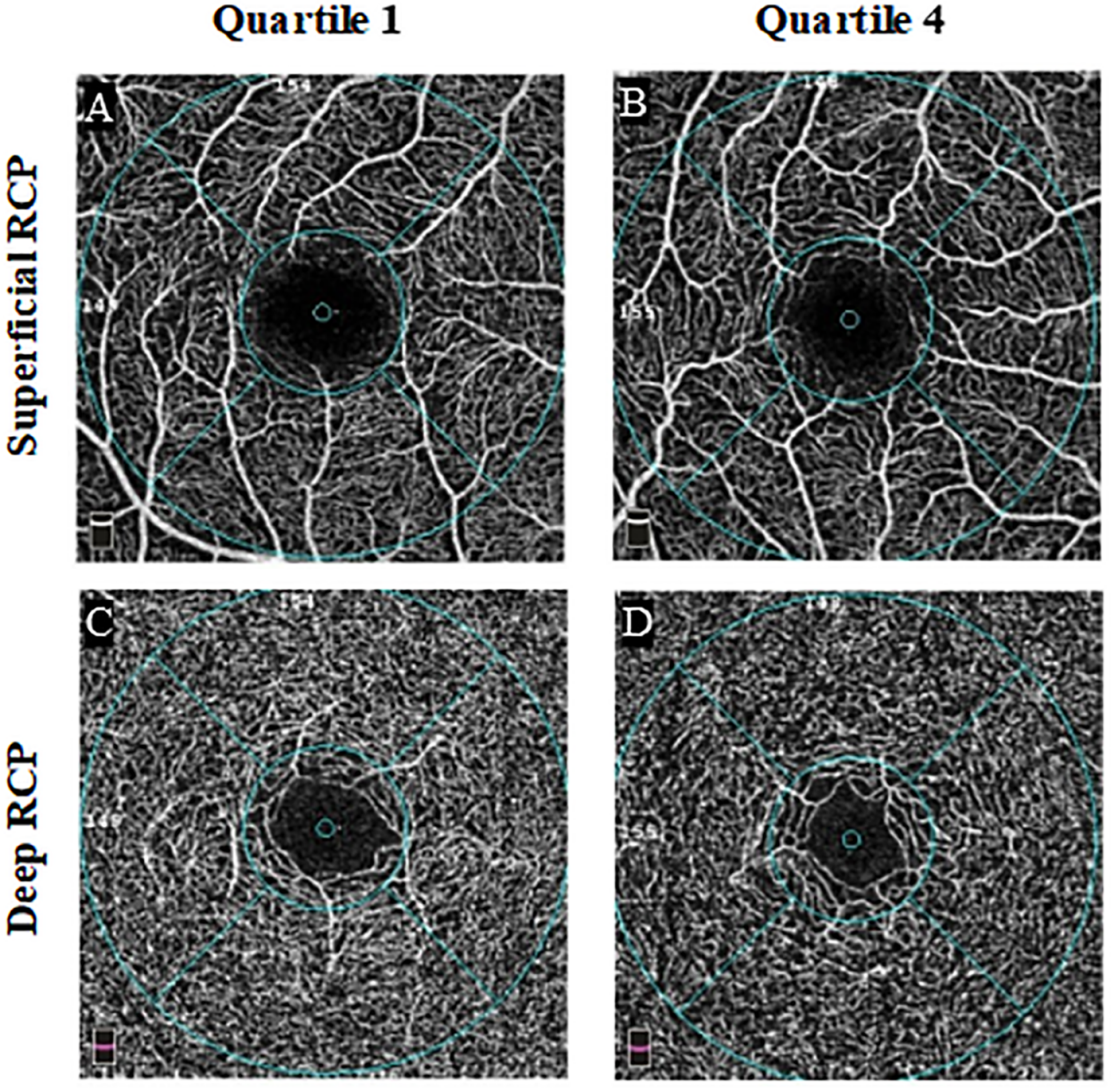
Representative OCTA images from Q1 and Q4. (**A**) Superficial RCP image from Q1. (**B**) Superficial RCP image from Q4. (**C**) Deep RCP image from Q1. (**D**) Deep RCP image from Q4. Deep RCP is sparser in (**D**) versus (**C**). Q1, RC < 0.68 mmol/L; Q4, RC ≥ 1.10 mmol/L.

The RCP characteristics in different RC quartile groups from the baseline survey are presented in Table 2. For the participants stratified into quartiles, the vessel density of the deep RCP decreased from 51.84% to 50.90% from Q1 to Q4 in the parafovea (*P* < 0.001). Vessel density data for other quadrants of the deep and superficial RCP are presented in Table 2. However, except for the nasal quadrant of the superficial RCP, we did not observe significant differences in the vessel density of the superficial RCP or the FAZ among the quartiles. Representative OCTA images of the participants in Q1 and Q4 are illustrated in Figure 2.

**Table 2.** Ocular Characteristics of Participants by RC Level Quartiles

	Mean ± SD						
Characteristic	Total	Q1	Q2	Q3	Q4	*P*	*P* for Trend
Superficial RCP (%)							
Fovea	15.82 ± 5.81	15.85 ± 5.70	15.69 ± 5.74	15.64 ± 5.90	16.11 ± 5.91	0.260	0.362
Parafovea	49.00 ± 3.20	49.11 ± 3.37	48.99 ± 3.33	49.04 ± 3.09	48.87 ± 3.00	0.391	0.132
Temporal	47.25 ± 3.23	47.34 ± 3.34	47.20 ± 3.36	47.34 ± 3.09	47.12 ± 3.13	0.344	0.259
Superior	50.43 ± 3.52	50.61 ± 3.68	50.36 ± 3.66	50.44 ± 3.42	50.33 ± 3.30	0.287	0.131
Nasal	48.21 ± 3.41	48.36 ± 3.61	48.27 ± 3.48	48.19 ± 3.32	48.03 ± 3.24	0.167	0.026
Inferior	50.12 ± 3.69	50.13 ± 3.87	50.15 ± 3.79	50.19 ± 3.62	50.00 ± 3.48	0.687	0.491
Deep RCP (%)							
Fovea	29.49 ± 7.06	29.76 ± 7.06	29.35 ± 6.99	29.15 ± 7.09	29.71 ± 7.08	0.180	0.733
Parafovea	51.46 ± 3.28	51.84 ± 3.21	51.64 ± 3.26	51.48 ± 3.26	50.90 ± 3.33	<0.001	<0.001
Temporal	51.82 ± 3.26	52.19 ± 3.12	52.01 ± 3.17	51.84 ± 3.22	51.24 ± 3.45	<0.001	<0.001
Superior	50.98 ± 3.73	51.39 ± 3.61	51.12 ± 3.76	50.98 ± 3.67	50.42 ± 3.81	<0.001	<0.001
Nasal	52.12 ± 3.27	52.47 ± 3.21	52.38 ± 3.21	52.08 ± 3.28	51.57 ± 3.32	<0.001	<0.001
Inferior	50.93 ± 3.82	51.31 ± 3.76	51.05 ± 3.79	51.00 ± 3.79	50.36 ± 3.87	<0.001	<0.001
FAZ (mm^2^)	0.33 ± 0.11	0.33 ± 0.12	0.33 ± 0.11	0.33 ± 0.11	0.32 ± 0.11	0.241	0.686

*P* values were calculated by ANOVA. *P* for trend was calculated with RC quartiles considered as a continuous ordinal variable. Participants were categorized into quartiles based on RC levels: Q1, RC < 0.68 mmol/L; Q2, 0.68 mmol/L ≤ RC < 0.88 mmol/L; Q3, 0.88 mmol/L ≤ RC < 1.10 mmol/L; Q4, RC ≥ 1.10 mmol/L.

### Association Between RC and the RCP

The associations between the RC and RCP vessel density are presented in Table 3. After adjusting for age, sex, current smoking status, current drinking status, hypertension status, diabetes status, BMI, AL, and CVD (Model 1), each 1-mmol/L increase in RC was associated with a 0.540 decrease in deep RCP vessel density in the parafovea (95% CI, −0.781 to −0.300; *P* < 0.001). The β values in the other four quadrants are shown in Table 3. For Model 2, after further adjustment for blood lipid parameters (TGs, LDL-C, and HDL-C) based on Model 1, the association between RC and the deep RCP remained significant (*P* < 0.001). Nevertheless, for superficial RCP vessel density or the FAZ, we observed no significant associations. Furthermore, we assessed the associations between RC quartiles and the RCP vessel density. As shown in Supplementary Table S1, compared with the lowest quartile group (Q1), the multivariate β values for Q2, Q3, and Q4 in the parafovea were −0.288 (95% CI, −0.555 to −0.020), −0.353 (95% CI, −0.621 to −0.084), and −0.552 (95% CI, −0.836 to −0.268), respectively (*P* for trend < 0.001), in Model 2. We observed similar associations in other regions of the deep RCP.

**Table 3.** Association Between Continuous RC and RCP

	Model 1		Model 2	
Characteristic	Adjusted β (95% CI)	*P*	Adjusted β (95% CI)	*P*
Superficial RCP				
Fovea	−0.157 (−0.599 to 0.286)	0.488	−0.136 (−0.682 to 0.409)	0.624
Parafovea	−0.122 (−0.373 to 0.129)	0.342	−0.274 (−0.583 to 0.036)	0.083
Temporal	−0.129 (−0.384 to 0.127)	0.324	−0.304 (−0.619 to 0.011)	0.058
Superior	−0.107 (−0.383 to 0.168)	0.445	−0.222 (−0.562 to 0.118)	0.200
Nasal	−0.219 (−0.488 to 0.051)	0.111	−0.415 (−0.747 to −0.084)	0.014
Inferior	−0.026 (−0.318 to 0.266)	0.862	−0.152 (−0.512 to 0.208)	0.408
Deep RCP				
Fovea	−0.507 (−1.041 to 0.027)	0.063	−0.335 (−0.993 to 0.324)	0.319
Parafovea	−0.540 (−0.781 to −0.300)	<0.001	−0.578 (−0.875 to −0.282)	<0.001
Temporal	−0.491 (−0.730 to −0.253)	<0.001	−0.543 (−0.837 to −0.249)	<0.001
Superior	−0.671 (−0.952 to −0.390)	<0.001	−0.683 (−1.030 to −0.337)	<0.001
Nasal	−0.501 (−0.740 to −0.262)	<0.001	−0.554 (−0.848 to −0.259)	<0.001
Inferior	−0.494 (−0.779 to −0.210)	<0.001	−0.529 (−0.880 to −0.179)	0.003
FAZ	0.006 (−0.003 to 0.015)	0.177	0.002 (−0.008 to 0.013)	0.666

The RC alterations were defined as per 1-mmol/L increase. Model 1 included age, sex, current smoking, current drinking, hypertension, diabetes, BMI, AL, and CVD. Model 2 included Model 1 plus TG, HDL-C, and LDL-C.

### Subgroup Analysis for the Relationship Between Continuous RC and the RCP

Supplementary Tables S2 to S4 show the associations between RC and RCP vessel density stratified by sex, hypertension status, and diabetes status. After adjusting for age, sex, current smoking status, current drinking status, hypertension status, diabetes status, BMI, AL, CVD, TG level, LDL-C level, and HDL-C level, a significant moderating effect of sex was observed on the association between RC and deep RCP vessel density in the parafoveal region (parafovea, *P* for interaction = 0.029; superior, *P* for interaction = 0.014; nasal, *P* for interaction = 0.015) (Supplementary Table S2), with a stronger negative correlation observed in males. No significant interaction effect of sex was observed on the associations between either the superficial RCP or the FAZ. Furthermore, we did not observe a moderating effect of hypertension or diabetes on the association between RC and RCP vessel density (all *P* values for interactions > 0.05) (Supplementary Tables S3, S4).

## Discussion

To our knowledge, this is the first study to evaluate the association between RC and RCP vessel density in a large community-based population using OCTA. Multivariable generalized linear model analysis revealed that higher RC levels were associated with lower deep RCP parafoveal vessel density, with deep RCP vessel density decreasing as the RC level increased. Moreover, we also identified a moderating effect of sex on the relationship between RC and deep RCP vessel density.

In our study, we showed that higher RC levels were associated with lower deep RCP vessel density in the parafovea, suggesting that RC may exert a damaging effect on the deep RCP. Previous studies have shown that dyslipidemia is significantly associated with decreased retinal vessel density in patients with chest pain^31^ and that dyslipidemia is independently associated with reduced retinal microvascular density in diabetic patients,^32^ which is consistent with the results of our study. Potentially, the decreased RCP in participants with higher RC levels may be attributed to endothelial injury caused by inflammation, oxidative stress, and endothelial cell dysfunction.^7^^,^^8^ The greater susceptibility of the deep RCP compared with the superficial RCP may be explained by the fact that the deep capillary plexus is comprised of more terminal vessels and exhibits higher metabolic demand, rendering it more vulnerable to the hemodynamic alterations and oxidative stress induced by dyslipidemia.^33^ However, further research is needed to explore these internal mechanisms.

As reported in prior studies, impairment of blood flow or hypoperfusion in the retinal vascular system leads to several dyslipidemia-related ocular diseases, such as DR and age-related macular degeneration.^17^^,^^34^^,^^35^ RC is recognized as a crucial risk factor for DR and plays a pivotal role in its pathogenesis. Aberrant accumulation of RC can lead to the detachment of RC particles, triggering local oxidative stress and inflammatory responses and subsequently resulting in microvascular ischemia and damage. These pathological changes may collectively contribute to the initiation and progression of DR.^9^ The findings of our study may help explain why individuals with higher RC levels are more prone to developing ocular diseases and visual impairment.^36^

In our study, no significant association was observed between RC levels and FAZ area. FAZ area enlargement is typically considered a late marker of widespread and sustained capillary loss leading to structural remodeling. Our participants were generally healthy without overt ocular diseases, representing a preclinical stage where RC-related microvascular changes may be too subtle to induce structural FAZ damage. Furthermore, the FAZ area exhibits substantial physiological variability even in individuals with normal vision, ranging from 0.071 to 0.527 mm^2^,^37^ which may mask potential mild effects of RC on the FAZ area.

A significant sex difference was observed in the association between RC levels and deep RCP vessel density in our study, with a stronger association detected in male participants. This finding indicates that, compared with female participants, the reduction in deep RCP vessel density in male participants is more susceptible to the impact of high RC levels, suggesting that sex may inform clinical risk stratification and screening strategies, with implications for closer ophthalmologic follow-up in males with elevated RC levels. This may be related to the damaging effect of androgens and the protective effect of estrogens on the retinal microvasculature.^38^^–^^40^ However, the association between RC levels and RCP vessel density was not influenced by hypertension or diabetes mellitus. Although previous studies have reported that hypertension and diabetes are associated with retinal microvascular damage,^26^^,^^27^ these interactions seem to be absent in our study. This might be attributed to regular use of antihypertensive and glucose-lowering medications among participants, which may have partially counteracted the microvascular damage otherwise caused by hypertension and diabetes, thereby attenuating their modifying effects in subgroup analyses. Nevertheless, these interaction effects may be influenced by chance factors and still must be verified through large-scale longitudinal studies and in other cohorts.

The major strengths of our study include the use of detailed ophthalmic examinations, standardized questionnaire information, biochemical analyses in a large community-based study, and adjustments for potential confounding factors and subgroup analyses to ensure the robustness of the results. However, this study has several limitations. First, due to the limitations of the cross-sectional design, we could not determine a causal relationship between RC levels and the RCP. Second, as a single-center study, the findings may not be generalizable to other regions, ethnic groups, or countries. Third, our OCTA imaging was confined to a 3 × 3 mm² macular area. Therefore, microvascular changes in the peripheral retina, which might also be affected by RC levels, could not be evaluated, and our findings may not be generalizable to the entire retinal vasculature. Fourth, compared with direct measurements, the RC values obtained indirectly through calculation formulas may lack precision, which may weaken the observed associations. Fifth, information on the use of lipid-lowering medications during the study period was not collected. According to previous studies, lipid-lowering medications may attenuate the association between RC and the retinal microvasculature,^36^^,^^41^^–^^43^ but our study cannot exclude the potential interference of these medications on the results.

## Conclusions

We found that higher RC levels were associated with lower deep RCP vessel density. Our findings help validate the detrimental effect of high RC levels on the retinal microvasculature, highlight the importance of controlling RC levels to prevent microvascular lesions, and provide a new perspective for future investigations into the microvascular mechanisms of cardiovascular diseases.

## Supplementary Material

Supplement 1
