# Supplementary material for: Association of Remnant Cholesterol With the Retinal Capillary Plexus
Source: Transl Vis Sci Technol. 2026 Apr 16;15(4):18. doi: 10.1167/tvst.15.4.18 (PMC13101856; doi:10.1167/tvst.15.4.18)
Supplement: Supplement 1 [file tvst-15-4-18_s001.docx]

**Supplementary Tables**

**Supplementary Table 1.** Association between RC and retinal capillary plexus.

**Supplementary Table 2.** Association between continuous RC and retinal capillary plexus with gender subgroup analysis.

**Supplementary Table 3.** Association between continuous RC and retinal capillary plexus with hypertension subgroup analysis.

**Supplementary Table 4.** Association between continuous RC and retinal capillary plexus with Diabetes subgroup analysis.

| **Supplementary Table 1. Association between RC and retinal capillary plexus** | | | | | | | | | | |  |
| --- | --- | --- | --- | --- | --- | --- | --- | --- | --- | --- | --- |
| **Characteristics** | **Adjusted β (95% CI) (Model 1)** | | | | **p  for Trend** | **Adjusted β (95% CI) (Model 2)** | | | | **p  for Trend** |  |
|  | **Q1** | **Q2** | **Q3** | **Q4** |  | **Q1** | **Q2** | **Q3** | **Q4** |  |  |
| **Superficial RCP** |  |  |  |  |  |  |  |  |  |  |  |
| Fovea | Ref | -0.026 (-0.516, 0.464) | -0.054 (-0.543, 0.435) | 0.024 (-0.468, 0.515) | 0.958 | Ref | -0.019 (-0.512, 0.474) | -0.039 (-0.533, 0.455) | 0.077 (-0.446, 0.600) | 0.818 |  |
| Parafovea | Ref | -0.157 (-0.435, 0.121) | -0.070 (-0.347, 0.208) | -0.175 (-0.454, 0.104) | 0.330 | Ref | -0.163 (-0.442, 0.117) | -0.083 (-0.364, 0.197) | -0.234 (-0.531, 0.063) | 0.199 |  |
| Temporal | Ref | -0.176 (-0.459, 0.107) | -0.004 (-0.286, 0.279) | -0.167 (-0.451, 0.117) | 0.473 | Ref | -0.182 (-0.467, 0.102) | -0.016 (-0.301, 0.269) | -0.232 (-0.534, 0.070) | 0.293 |  |
| Superior | Ref | -0.291 (-0.596, 0.015) | -0.148 (-0.452, 0.157) | -0.175 (-0.481, 0.131) | 0.434 | Ref | -0.290 (-0.596, 0.017) | -0.154 (-0.462, 0.154) | -0.213 (-0.539, 0.112) | 0.326 |  |
| Nasal | Ref | -0.128 (-0.426, 0.170) | -0.174 (-0.471, 0.124) | -0.282 (-0.580, 0.017) | 0.065 | Ref | -0.143 (-0.442, 0.157) | -0.192 (-0.492, 0.109) | -0.353 (-0.671, -0.035) | 0.032 |  |
| Inferior | Ref | -0.035 (-0.358, 0.289) | 0.051 (-0.272, 0.374) | -0.072 (-0.396, 0.252) | 0.805 | Ref | -0.038 (-0.363, 0.287) | 0.033 (-0.293, 0.359) | -0.137 (-0.481, 0.208) | 0.567 |  |
| **Deep RCP** |  |  |  |  |  |  |  |  |  |  |  |
| Fovea | Ref | -0.245 (-0.837, 0.347) | -0.398 (-0.989, 0.192) | -0.308 (-0.901, 0.285) | 0.258 | Ref | -0.216 (-0.811, 0.378) | -0.323 (-0.919, 0.273) | -0.118 (-0.749, 0.513) | 0.616 |  |
| Parafovea | Ref | -0.306 (-0.572, -0.039) | -0.387 (-0.652, -0.121) | -0.604 (-0.871, -0.337) | <0.001 | Ref | -0.288 (-0.555, -0.020) | -0.353 (-0.621, -0.084) | -0.552 (-0.836, -0.268) | <0.001 |  |
| Temporal | Ref | -0.271 (-0.535, -0.006) | -0.353 (-0.617, -0.090) | -0.605 (-0.870, -0.340) | <0.001 | Ref | -0.255 (-0.520, 0.010) | -0.324 (-0.590, -0.058) | -0.573 (-0.855, -0.291) | <0.001 |  |
| Superior | Ref | -0.389 (-0.701, -0.078) | -0.465 (-0.776, -0.154) | -0.681 (-0.993, -0.368) | <0.001 | Ref | -0.360 (-0.673, -0.047) | -0.415 (-0.729, -0.101) | -0.589 (-0.921, -0.257) | <0.001 |  |
| Nasal | Ref | -0.189 (-0.454, 0.075) | -0.399 (-0.663, -0.135) | -0.548 (-0.813, -0.282) | <0.001 | Ref | -0.184 (-0.450, 0.082) | -0.375 (-0.642, -0.108) | -0.510 (-0.792, -0.228) | <0.001 |  |
| Inferior | Ref | -0.372 (-0.687, -0.057) | -0.330 (-0.645, -0.016) | -0.579 (-0.895, -0.263) | <0.001 | Ref | -0.351 (-0.667, -0.034) | -0.299 (-0.616, 0.019) | -0.532 (-0.869, -0.196) | 0.004 |  |
| **FAZ** | Ref | 0.003 (-0.007, 0.013) | 0.004 (-0.006, 0.013) | 0.004 (-0.006, 0.014) | 0.421 | Ref | 0.002 (-0.007, 0.012) | 0.003 (-0.007, 0.012) | 0.001 (-0.009, 0.011) | 0.834 |  |
| p for trend was tested with generalized linear models by considering the RC quartiles as an ordinal variable.  Participants were categorized into quartiles based on RC levels. Q1 (n=976): RC < 0.68 mmol/L; Q2 (n=966): 0.68 mmol/L ≤ RC < 0.88 mmol/L; Q3 (n=978): 0.88 mmol/L ≤ RC < 1.10 mmol/L; Q4 (n=983): RC ≥ 1.10 mmol/L. Model 1: age, sex, current smoking, current drinking, hypertension, diabetes, BMI, AL, and CVD.  Model 2: Model 1 + TG, HDL-C, and LDL-C.  Ref, reference; RC, remnant cholesterol; BMI, body mass index; AL, axial length; RCP, retinal capillary plexus; FAZ, foveal avascular zone; CVD, cardiovascular disease. | | | | | | | | | | |  |
|  |  |  |  |  |  |  |  |  |  |  |  |
|  |  |  |  |  |  |  |  |  |  |  |  |

| **Supplementary Table 2. Association between continuous RC and retinal capillary plexus with gender subgroup analysis.** | | | |  |
| --- | --- | --- | --- | --- |
| **Characteristics** | **Male（n=1957）** | **Female（n=1946）** | **p for  interaction** |  |
|  | **Adjusted β（95&CI)** | **Adjusted β（95&CI)** |  |  |
| **Superficial RCP** |  |  |  |  |
| Fovea | -0.135 (-0.873, 0.604) | -0.163 (-0.999, 0.674) | 0.812 |  |
| Parafovea | -0.383 (-0.825, 0.059) | -0.136 (-0.584, 0.313) | 0.630 |  |
| Temporal | -0.418 (-0.861, 0.025) | -0.158 (-0.622, 0.306) | 0.555 |  |
| Superior | -0.354 (-0.834, 0.127) | -0.086 (-0.584, 0.411) | 0.709 |  |
| Nasal | -0.522 (-0.991, -0.054) | -0.291 (-0.778, 0.196) | 0.608 |  |
| Inferior | -0.234 (-0.750, 0.283) | -0.006 (-0.525, 0.512) | 0.750 |  |
| **Deep RCP** |  |  |  |  |
| Fovea | -0.463 (-1.308, 0.381) | -0.219 (-1.275, 0.838) | 0.763 |  |
| Parafovea | -0.861 (-1.285, -0.437) | -0.278 (-0.705, 0.149) | 0.029 |  |
| Temporal | -0.757 (-1.169, -0.345) | -0.333 (-0.767, 0.101) | 0.070 |  |
| Superior | -1.050 (-1.538, -0.561) | -0.269 (-0.777, 0.240) | 0.014 |  |
| Nasal | -0.890 (-1.310, -0.471) | -0.219 (-0.648, 0.209) | 0.015 |  |
| Inferior | -0.741 (-1.240, -0.242) | -0.287 (-0.797, 0.223) | 0.170 |  |
| **FAZ** | 0.004 (-0.010, 0.017) | 0.001 (-0.017, 0.018) | 0.696 |  |
| Adjusted for age, sex, current smoking, current drinking, hypertension, diabetes, BMI, AL, CVD, TG, HDL-C, and LDL-C.  The RC alterations were defined as per 1 mmol/L increase. Interaction effect was calculated from models that included interaction terms of factor x RC, and were adjusted for age, sex, current smoking, current drinking, hypertension, diabetes, BMI, AL, CVD, TG, HDL-C, and LDL-C. RC, remnant cholesterol; BMI, body mass index; AL, axial length; RCP, retinal capillary plexus; FAZ, foveal avascular zone; CVD, cardiovascular disease. | | | |  |
|  |  |  |  |  |
|  |  |  |  |  |
|  |  |  |  |  |
|  |  |  |  |  |
|  |  |  |  |  |
|  |  |  |  |  |

| **Supplementary Table 3. Association between continuous RC and retinal capillary plexus with hypertension subgroup analysis.** | | | |  |
| --- | --- | --- | --- | --- |
| **Characteristics** | **Hypertension（n=925）** | **Non-Hypertension（n=2978）** | **p for  interaction** |  |
|  | **Adjusted β（95&CI)** | **Adjusted β（95&CI)** |  |  |
| **Superficial RCP** |  |  |  |  |
| Fovea | -0.829 (-1.890, 0.233) | 0.125 (-0.513, 0.762) | 0.419 |  |
| Parafovea | -0.063 (-0.691, 0.565) | -0.339 (-0.695, 0.018) | 0.486 |  |
| Temporal | -0.459 (-1.086, 0.169) | -0.240 (-0.606, 0.125) | 0.807 |  |
| Superior | 0.090 (-0.621, 0.802) | -0.315 (-0.702, 0.072) | 0.329 |  |
| Nasal | -0.187 (-0.853, 0.479) | -0.499 (-0.883, -0.116) | 0.555 |  |
| Inferior | 0.306 (-0.428, 1.039) | -0.299 (-0.712, 0.115) | 0.262 |  |
| **Deep RCP** |  |  |  |  |
| Fovea | -0.838 (-2.105, 0.429) | -0.107 (-0.879, 0.664) | 0.433 |  |
| Parafovea | -0.048 (-0.647, 0.552) | -0.740 (-1.081, -0.398) | 0.314 |  |
| Temporal | 0.141 (-0.453, 0.735) | -0.756 (-1.095, -0.417) | 0.188 |  |
| Superior | -0.224 (-0.916, 0.467) | -0.823 (-1.224, -0.422) | 0.851 |  |
| Nasal | -0.224 (-0.844, 0.395) | -0.656 (-0.991, -0.321) | 0.432 |  |
| Inferior | 0.128 (-0.570, 0.825) | -0.722 (-1.128, -0.315) | 0.141 |  |
| **FAZ** | 0.005 (-0.016, 0.026) | 0.001 (-0.012, 0.013) | 0.810 |  |
| Adjusted for age, sex, current smoking, current drinking, hypertension, diabetes, BMI, AL, CVD, TG, HDL-C, and LDL-C.  The RC alternations were defined as per 1 mmol/L increase. Interaction effect was calculated from models that included interaction terms of factor x RC, and were adjusted for age, sex, current smoking, current drinking, hypertension, diabetes, BMI, AL, CVD, TG, HDL-C, and LDL-C. RC, remnant cholesterol; BMI, body mass index; AL, axial length; RCP, retinal capillary plexus; FAZ, foveal avascular zone; CVD, cardiovascular disease. | | | |  |
|  |  |  |  |  |
|  |  |  |  |  |
|  |  |  |  |  |
|  |  |  |  |  |
|  |  |  |  |  |
|  |  |  |  |  |

| **Supplementary Table 4. Association between continuous RC and retinal capillary plexus with Diabetes subgroup analysis.** | | | |  |
| --- | --- | --- | --- | --- |
| **Characteristics** | **Diabetes（n=360）** | **Non-Diabetes（n=3543）** | **p for  interaction** |  |
|  | **Adjusted β（95&CI)** | **Adjusted β（95&CI)** |  |  |
| **Superficial RCP** |  |  |  |  |
| Fovea | -0.586 (-1.801, 0.628) | -0.012 (-0.631, 0.608) | 0.251 |  |
| Parafovea | -0.214 (-0.962, 0.534) | -0.284 (-0.633, 0.065) | 0.941 |  |
| Temporal | -0.204 (-0.951, 0.543) | -0.309 (-0.664, 0.047) | 0.541 |  |
| Superior | -0.174 (-1.005, 0.656) | -0.222 (-0.604, 0.160) | 0.859 |  |
| Nasal | -0.430 (-1.219, 0.360) | -0.428 (-0.802, -0.054) | 0.701 |  |
| Inferior | -0.048 (-0.891, 0.796) | -0.175 (-0.581, 0.232) | 0.931 |  |
| **Deep RCP** |  |  |  |  |
| Fovea | -0.419 (-1.894, 1.056) | -0.312 (-1.060, 0.435) | 0.592 |  |
| Parafovea | -0.466 (-1.148, 0.216) | -0.606 (-0.942, -0.271) | 0.514 |  |
| Temporal | -0.307 (-0.988, 0.374) | -0.576 (-0.908, -0.243) | 0.876 |  |
| Superior | -0.633 (-1.415, 0.149) | -0.695 (-1.088, -0.302) | 0.654 |  |
| Nasal | -0.513 (-1.217, 0.191) | -0.587 (-0.919, -0.255) | 0.251 |  |
| Inferior | -0.400 (-1.184, 0.383) | -0.566 (-0.965, -0.168) | 0.498 |  |
| **FAZ** | -0.003 (-0.028, 0.022) | 0.003 (-0.009, 0.015) | 0.637 |  |
| Adjusted for age, sex, current smoking, current drinking, hypertension, diabetes, BMI, AL, CVD, TG, HDL-C, and LDL-C.  The RC alternations were defined as per 1 mmol/L increase. Interaction effect was calculated from models that included interaction terms of factor x RC, and were adjusted for age, sex, current smoking, current drinking, hypertension, diabetes, BMI, AL, CVD, TG, HDL-C, and LDL-C. RC, remnant cholesterol; BMI, body mass index; AL, axial length; RCP, retinal capillary plexus; FAZ, foveal avascular zone; CVD, cardiovascular disease. | | | |  |
|  |  |  |  |  |
|  |  |  |  |  |
|  |  |  |  |  |
|  |  |  |  |  |
|  |  |  |  |  |
|  |  |  |  |  |
